# Brain MRI findings in patients with post COVID-19 condition: frequency and longitudinal changes in a nationwide cohort study

**DOI:** 10.3389/fneur.2025.1662263

**Published:** 2025-11-13

**Authors:** Liv Lygre Furevik, Oksana Lapina, Elisabeth Stokke Lindland, Einar August Høgestøl, Oliver Marcel Geier, Kristina Devik, Anette Huuse Farmen, Heidi Øyen Flemmen, Hanne Flinstad Harbo, Åse Hagen Morsund, Vojtech Novotny, Hilde Karen Ofte, Kenneth Ottesen Pedersen, Trine Haug Popperud, Barbara Ratajczak-Tretel, Christian Samsonsen, Per Selnes, Øivind Torkildsen, Ragnhild Marie Undseth, Anne Hege Aamodt, Mona Kristiansen Beyer, Marion Ingeborg Boldingh

**Affiliations:** 1 Division of Radiology and Nuclear Medicine, Oslo University Hospital, Oslo, Norway; 2 Faculty of Medicine, Institute of Clinical Medicine, University of Oslo, Oslo, Norway; 3 Department of Radiology, Sorlandet Hospital, Arendal, Norway; 4 Department of Neurology, Oslo University Hospital, Oslo, Norway; 5 Department of Psychology, Center for Lifespan Changes in Brain and Cognition, University of Oslo, Oslo, Norway; 6 Department of Neurology, Namsos Hospital, Namsos, Norway; 7 Department of Neurology, Innlandet Hospital Trust, Lillehammer, Norway; 8 Department of Neurology, Telemark Hospital, Skien, Norway; 9 Department of Neurology, Molde Hospital, Molde, Norway; 10 Department of Neurology, Nordland Hospital Trust, Bodø, Norway; 11 The Intervention Centre, Oslo University Hospital, Oslo, Norway; 12 Department of Neurology, Østfold Hospital Trust, Grålum, Norway; 13 Department of Neurology and Clinical Neurophysiology, St. Olav’s Hospital, Trondheim, Norway; 14 Department of Neuromedicine and Movement Science, Faculty of Medicine and Health Sciences, Norwegian University of Science and Technology (NTNU), Trondheim, Norway; 15 Department of Neurology, Akershus University Hospital, Lørenskog, Norway; 16 Department of Neurology, Haukeland University Hospital, Bergen, Norway; 17 Department of Clinical Medicine, University of Bergen, Bergen, Norway; 18 Faculty of Health and Life Sciences, Institute of Population Health, University of Liverpool, Liverpool, United Kingdom

**Keywords:** post COVID-19 condition, long COVID, brain MRI, neuroimaging, neurological symptoms, cranial nerve enhancement, longitudinal

## Abstract

**Background:**

Prolonged neurological symptoms following COVID-19 are common, yet few longitudinal studies describe brain MRI findings in this patient group. The use of contrast enhanced sequences is particularly lacking. We address this knowledge gap by reporting the frequency and longitudinal changes in brain MRI findings among patients with post COVID-19 condition exhibiting neurological symptoms.

**Methods:**

This prospective multicenter study included 140 adult patients referred for persistent neurological symptoms following COVID-19. Brain MRI was performed at both 6 and 12 months after infection onset, reporting white matter hyperintensities, cerebral microbleeds, and additional pathological findings including contrast enhancement. White matter hyperintensities were compared with a healthy control group.

**Results:**

The prevalence of white matter hyperintensities was comparable to healthy controls, and microbleeds were found at rates comparable to population studies, with longitudinal changes being infrequent. Lesions consistent with inflammation or demyelination were present in 4% (5/120) of patients at 6 months. Cranial nerve enhancement was found in 7% (7/94) of patients, persisting up to 12 months, predominantly affecting the oculomotor nerve. However, enhancement occurred without clinically detected ocular muscle paresis.

**Conclusion:**

Our findings indicate that brain MRI primarily serves to exclude differential diagnoses in post COVID-19 condition, with limited clinical benefit of repeated imaging in the absence of new symptoms. However, signs of long-term inflammatory processes can be observed, and detection is improved by contrast enhanced sequences.

## 1 Introduction

The emergence of long COVID, formally termed post COVID-19 condition (PCC), highlights the persistent post-infectious symptoms experienced by approximately 10% of adults who have contracted COVID-19 (1, 2). According to the World Health Organization, PCC is defined as symptoms that start within 3 months of a SARS-CoV-2 infection, persist for at least 2 months, and cannot be explained by another condition (3). Neurological and neuropsychiatric symptoms such as cognitive impairment, headaches, sleep disturbances, anosmia/hyposmia, and fatigue are among the most common complaints (4).

Brain MRI serves as a valuable tool for detecting abnormalities that may explain neurological or cognitive symptoms in individuals with PCC and excluding other potential causes. Despite the significant number of people experiencing neurological symptoms associated with PCC, comprehensive studies examining routine brain MRI findings, such as structural changes or signs of inflammation, are sparse and yield inconsistent results. A scoping review from July 2023 identified only seven relevant studies comprising a total of 451 participants, with only six participants undergoing imaging at multiple time points, highlighting the scarcity of longitudinal data (5). The most common MRI findings were perivascular spaces (PVS), cerebral microbleeds (CMBs), and white matter hyperintensities (WMHs). These non-specific changes are not unique to PCC, and their frequency differed considerably across studies, offering limited utility for the research findings. Notably, none of the studies included sequences with intravenous contrast agents, limiting the detection of inflammatory changes and leaving significant gaps in our understanding. Additionally, 53% (240/451) of the participants were hospitalized during their infection, with many requiring intensive care unit (ICU) treatment. This may introduce bias due to the underrepresentation of non-hospitalized participants, who constitute the majority of people with PCC (1). PCC study populations are heterogeneous, including participants with and without neurological symptoms, as well as those with symptoms from other organ systems, such as the cardiovascular or respiratory systems.

The disparity and lack of existing data present challenges for clinicians and radiologists in determining the appropriate indications and methods for diagnostic imaging when assessing PCC patients with neurological symptoms. A global expert consensus advises to perform brain MRI but lacks specific recommendations regarding sequence selection and the use of intravenous contrast agents (6). Consequently, findings from longitudinal studies examining brain MRI in this patient group are essential for developing guidelines. Our study aims to address these gaps by reporting the frequency and longitudinal changes in brain MRI findings among patients with persistent neurological complaints 6 months after COVID-19, in a cohort where the majority were not hospitalized during infection.

## 2 Methods

### 2.1 Study design

The Norwegian NeuroCOVID (NNC) study is a prospective, observational, multicenter study assessing patients referred to neurology departments for persistent neurological symptoms after COVID-19. For a targeted analysis of WMHs, healthy individuals with pre-pandemic MRI scans serve as a control group. The study received approval from the South-Eastern Norway Regional Committee for Medical Research Ethics (no. 152727) and institutional data protection services and was registered *a priori* with ClinicalTrials.gov (NCT04576351). The study was conducted in accordance with the Declaration of Helsinki. All participants provided written informed consent.

### 2.2 Participant selection

**Figure 1:**
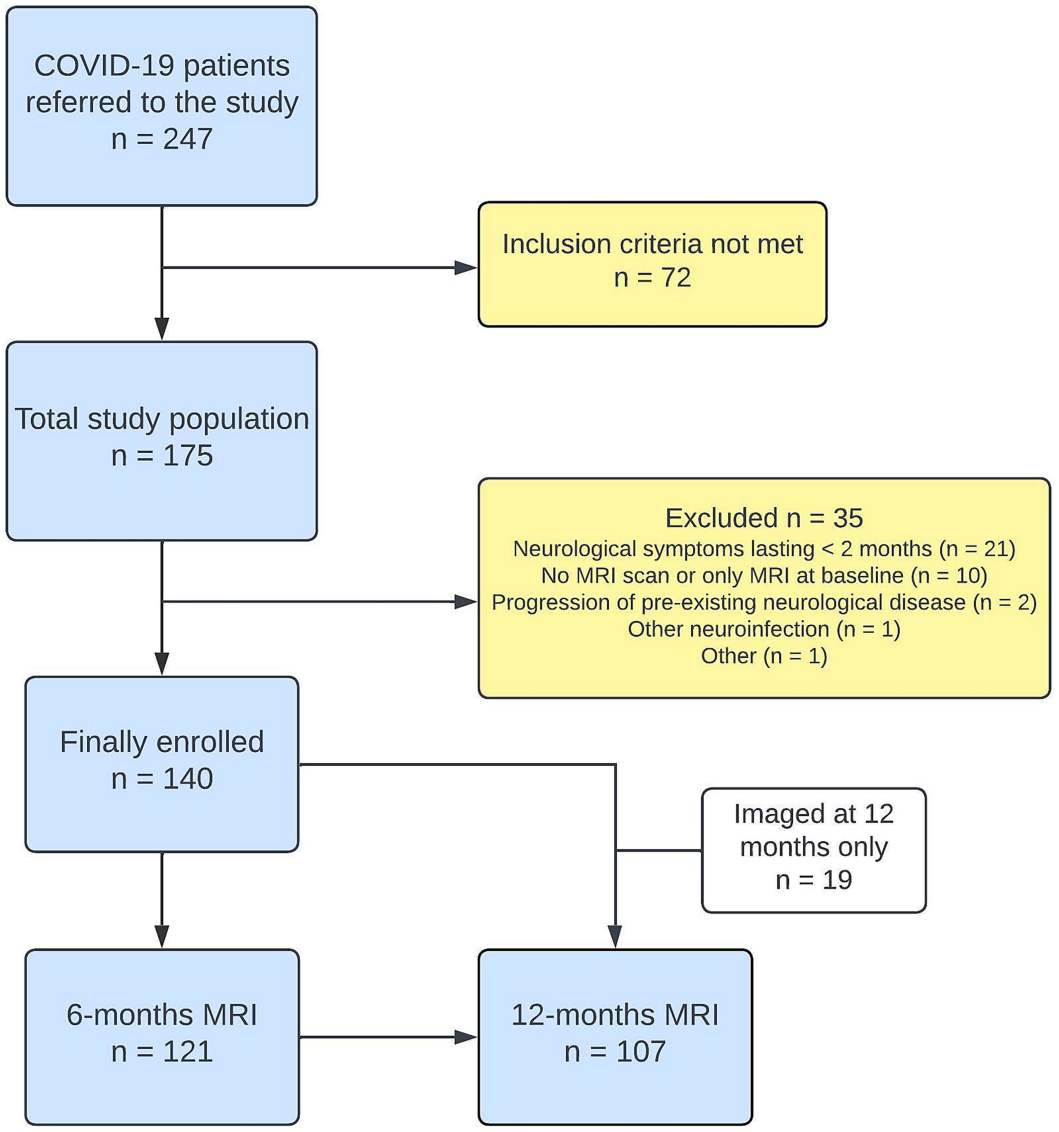
Flowchart showing inclusion and follow-up of PCC participants. Clinical evaluations were conducted concurrently with MRI examinations. The control group (*n* = 64) was derived from a prior study, detailed in the Methods section.

Between April 2020 and June 2023, adults above 18 years of age who developed neurological, neuropsychological, or neuropsychiatric symptoms temporally linked to a confirmed SARS-CoV-2 infection—verified via polymerase chain reaction (PCR) or antibody testing—were recruited from 10 neurological departments across Norway. Referrals were made by general practitioners or other medical specialists. Participants were included in this dataset only if their symptoms persisted for more than 2 months after COVID-19 onset and they completed MRI at least once during either the 6- or 12-month follow-up. Additional reasons for exclusion are provided in Figure 1. The control group for WMH comparison comprises healthy volunteers from a prior study conducted between August 2016 and March 2019 (7), all of whom provided renewed consent for inclusion.

### 2.3 Clinical and laboratory data collection

Participants underwent comprehensive evaluations by a neurologist at 6 and 12 months after infection, including a standardized neurological examination and other assessments, with results from the latter reported in manuscripts currently under review. Demographic characteristics and comorbidities were collected, alongside information about neurological manifestations during the acute phase of the infection. COVID-19 vaccination status was obtained from the Norwegian Immunization Registry. Cerebrospinal fluid (CSF) samples were collected by lumbar puncture at the 6- or 12-month follow-up as a supplementary examination when clinically required. Indications for lumbar puncture included ongoing neurological symptoms, such as chronic headache, or MRI findings suggesting inflammation. Brain MRIs were performed using MRI scanners located at each participating center.

### 2.4 Brain imaging protocol and outcome measures

The study employed a standardized MRI protocol to accommodate scanners from two different MRI system vendors. Specific protocol details are available in Supplementary Table 1. Two neuroradiologists independently assessed the MRI scans for the PCC group; both were blinded to clinical data and each other’s evaluations. The first rater (LLF) interpreted all examinations, whereas the second rater (OL) interpreted those from the main recruitment center, encompassing 80 out of 140 participants.

Nonspecific WMHs on T2-weighted fluid attenuated inversion recovery (FLAIR) images were graded using the commonly used Fazekas scale (8), ranging from 0 to 3 based on the size and confluence of WMHs located in the deep white matter, and with lesion count. The lesion count method categorized WMHs into intervals: No lesions, 1–9 lesions, 10–20 lesions, and >20 lesions (9), with the exact number also recorded. For the PCC group, lesion counts were performed by rater LLF, while a third neuroradiologist (ESL) assessed this for the control group. Both LLF and ESL applied the Fazekas scale in controls.

CMBs, defined as small (<10 mm) intracerebral hemorrhages identified on hemorrhage-sensitive sequences (10), primarily susceptibility-weighted imaging (SWI), were noted if ≥1 was visually detected. The occurrence of new CMBs during follow-up, as well as the total number of CMBs for each participant, was recorded.

Pathological gadolinium enhancement of the meninges, brain parenchyma, cranial nerves (CN), and vessel walls was registered (yes/no), alongside any additional findings. For vessel wall analysis, a dedicated contrast enhanced T1-space black-blood imaging sequence was employed.

### 2.5 Statistical analysis

Statistical analyses were performed using IBM SPSS Statistics (version 30). Interrater reliability was assessed with Cohen’s Kappa for CMBs and contrast enhancement, and Cohen’s Weighted Kappa for Fazekas score. For group comparisons, normally distributed continuous variables were analyzed with independent *t*-tests, while categorical variables used chi-squared or Fisher’s exact tests. Binary matched-pairs data were analyzed using the McNemar mid-*p* test, and ordinal matched-pairs data using the Wilcoxon signed-rank test. The Mann–Whitney *U* test was used for analyzing WMHs between the PCC and control groups, and for analyzing differences in MRI time intervals between groups. Adjusted *p*-values were derived from ordinal logistic regression with a logit link, incorporating age as a covariate. Statistical significance was set at a two-sided *p*-value of 0.05.

## 3 Results

### 3.1 Participant characteristics

Of the 175 participants assessed for eligibility, 140 were included in the final analysis (Figure 1). The study population consisted of 59% females (83/140). The mean age was 46.7 years (SD = 13.8 years, range 18–83 years). Hospitalization was required for 41% (57/140) of participants during their infection, including 11% (15/140) admitted to the ICU. Detailed demographic and baseline data are presented in Table 1. Compared to non-hospitalized participants, those hospitalized were older and had more comorbidities such as hypertension and diabetes. Acute neurological symptoms, including ischemic stroke and encephalitis, manifested in 16% (23/140) of participants during the infection. The remaining 84% (117/140) experienced less acute manifestations, such as cognitive impairment or persistent headache. Six-month clinical assessments for 128 participants revealed predominant symptoms like fatigue (71%), cognitive impairment (65%), hyposmia (49%), and persistent headache (43%). A total of 24% (34/140) of participants underwent lumbar puncture during follow-up, on average 10.7 (SD = 4.3) months after infection onset. The control group, consisting of 64 individuals, 55% females (35/64), had a mean age of 57.5 years (SD = 12.9 years, range 26–81 years). While both groups had similar sex distributions (*p* = 0.54), analyses were adjusted for age due to significant differences, with the control group being older (mean difference 9.8 years, 95% CI 5.8–13.8, *p* < 0.001).

**Table 1:** Demographic and clinical characteristics of patients stratified by disease severity.

Variables	All patients *n* = 140	Outpatients *n* = 83	Hospitalized *n* = 57	*p*-value
Sex, female, *n* (%)	83 (59.3)	55 (66.3)	28 (49.1)	0.043*
Age, years, mean (SD)	46.7 (13.8)	42.5 (12.7)	52.7 (13.1)	<0.001*
ICU admission	15 (10.7)	0 (0)	15 (26.3)	
Current smoker, *n* (%)	8 (5.7)	6 (7.2)	2 (3.5)	0.352
Vaccinated before COVID-19 onset, *n* (%)	33 (23.6)	25 (30.1)	8 (14.0)	0.028*
Hypertension, *n* (%)	26 (18.6)	5 (6.0)	21 (36.8)	<0.001*
Diabetes, *n* (%)	6 (4.3)	0 (0)	6 (10.5)	0.003*
Cardiovascular disease, *n* (%)	7 (5.0)	1 (1.2)	6 (10.5)	0.013*
Kidney disease, *n* (%)	1 (0.7)	0 (0)	1 (1.8)	0.226
Asthma or COPD, *n* (%)	25 (17.9)	12 (14.5)	13 (22.8)	0.205
Malignancy, *n* (%)	3 (2.1)	0 (0)	3 (5.3)	0.035*

^*^Denotes statistical significance. COPD, chronic obstructive pulmonary disease.

### 3.2 Brain MRI findings and longitudinal changes

MRI scans at 6- and 12-month follow-ups were conducted in 86% (121/140) and 76% (107/140) of participants, respectively, at a median (interquartile range) of 201 (174–244) days and 377 (358–430) days since COVID-19 symptom onset. Overall, 63% (88/140) underwent MRI at both 6- and 12-month follow-ups. An intravenous contrast agent was administered to 80% (112/140) of participants at any time point, with 54% (75/140) receiving it at both 6 and 12 months. In comparison, 97% (62/64) of the control group completed MRI scans at two time points, with a median interval of 210 (196–217) days between scans, while the corresponding interval for the PCC group was 182 (168–196) days (*p* < 0.001). Interrater reliability for assessments of WMHs, CMBs, and contrast enhancement is presented in Supplementary Table 2, showing substantial (kappa = 0.61–0.80) to almost perfect (kappa = 0.81–1.00) agreement (11).

MRI findings are listed in Table 2, with further details on WMHs in PCC and control groups provided in Supplementary Table 3. Collected findings from 6- and 12-month MRIs showed that most PCC participants had Fazekas scores of 0 or 1, while 8% (11/139) had scores of 2 or 3. The Fazekas score did not significantly differ from the control group (*p* = 0.128). Comparing WMH lesion count score between groups revealed controls had more WMHs (*p* < 0.001 at 6 months); however, this difference disappeared after adjusting for age (*p* = 0.295). CMBs were present in a total of 16% (21/131) of PCC participants, with 4% (5/131) having more than 3 CMBs. There was a trend toward a higher lesion burden in hospitalized individuals (*p* = 0.052 for WMH lesion count score and *p* = 0.036 for CMBs).

**Table 2:** Pathological MRI findings at 6 and 12 months after infection.

MRI findings	6 months (*n* = 121)	12 months (*n* = 107)
**WMH lesion count^a^**		
0	56 (46.7)	50 (46.7)
1	50 (41.7)	44 (41.1)
2	7 (5.8)	7 (6.5)
3	7 (5.8)	6 (5.6)
**CMB**		
CMB > 0^b^	18 (16.2)	16 (15.5)
CMB > 3	5 (4.5)	3 (2.9)
**Inflammatory lesion**	5 (4.2)	4 (3.8)
**Contrast enhancement^c^** ^c^		
Meninges	0 (0)	0 (0)
Parenchyma	0 (0)	1 (1.1)
Cranial nerves	5 (5.4)	7 (7.4)
Vessel wall	0 (0)	0 (0)
**Incidental findings**		
Sinusitis	5 (4.1)	5 (4.7)
Global cortical atrophy	9 (7.4)	7 (6.5)
Lacunes/infarcts	5 (4.1)	5 (4.7)
Other^d^	14 (11.6)	15 (14.0)

Values in cells indicate the number (%) of participants with the corresponding MRI finding.

No statistically significant differences were observed between MRI findings at 6 and 12 months (all *p*-values > 0.05).

a 120/121 and 107/107 with T2 FLAIR imaging at 6 and 12 months, respectively.

b 111/121 and 103/107 with hemorrhage-sensitive sequences at 6 and 12 months, respectively. 123/131 imaged with SWI, 8/131 imaged with T2^*^-weighted gradient-recalled echo imaging.

c 93/121 and 94/107 with enhanced imaging at 6 and 12 months, respectively.

d Arterial aneurism (4), meningioma (4), cavernous malformations (3), premorbid demyelinating lesions (1), hemosiderosis (1), schwannoma (1), Chiari I (1).

At 6 months, distinct high intensity lesions on native (non-contrast) T2 FLAIR images, consistent with inflammation and/or demyelination, were identified in 4% (5/120) of participants. Notable findings included one participant with trigeminal symptoms exhibiting hyperintensity along the pontine segment of CN V. Two participants showed signs indicative of prior encephalitis, manifested as persistent hyperintensities: one in the hippocampus, and the other in the splenium, pons, and medulla oblongata. Two participants had lesions with a pattern suggestive of demyelination, one of which initially displayed a pattern interpreted as acute disseminated encephalomyelitis (ADEM). Additionally, hemorrhages in the thalamus and hippocampus, likely resulting from acute necrotizing encephalopathy, were noted in one participant.

Cranial nerve enhancement (CNE) was observed in 5% (5/93) of the participants receiving intravenous contrast at 6 months, predominantly bilateral in nature (80%), and most often affecting CN III (50%). Enhancement was also recorded in CN VII and CN VIII, with 3% (3/93) showing enhancement in multiple nerves. One participant with sensorineural hearing loss exhibited enhancement of CN VIII. However, enhancement of CN III and CN VII was subclinical, as there were no signs of ocular muscle paresis or facial weakness in these participants. Except for one instance of nonspecific punctate parenchymal contrast enhancement at 12 months, no parenchymal, meningeal, or vessel wall enhancement was observed at 6 or 12 months.

Fazekas scores remained stable across the 6- and 12-month evaluations in all 88 PCC participants undergoing T2 FLAIR imaging at both intervals, as well as in all 62 controls. Within the PCC group, 5% (4/88) developed one new WMH lesion, while another 5% (4/88) showed a reduction of one lesion. The proportion of participants exhibiting any change was lower in the PCC group compared to the control group; however, this difference was not statistically significant (*p* = 0.31). Newly detected CMBs were identified in 4% (3/83) of participants on repeated hemorrhage-sensitive sequences at 12 months, with only one participant presenting their initial CMB. No new ischemic infarctions were identified during the follow-up period.

Among the lesions initially interpreted as encephalitic, the hippocampal lesion resolved by 12 months, whereas the lesions in the splenium, pons, and medulla oblongata persisted. The native signal increase in CN V also persisted. Both participants with lesions suggestive of demyelination showed disease progression, and oligoclonal bands were detected in their CSF samples collected during this study, resulting in the diagnoses of multiple sclerosis (MS). Except for the previously mentioned oligoclonal bands, CSF samples obtained from all five participants with lesions interpreted as inflammatory or demyelinating were otherwise normal (white blood cells <4 × 10^9^/L; mean total protein 0.37 g/L, range 0.24–0.48 g/L).

**Figure 2:**
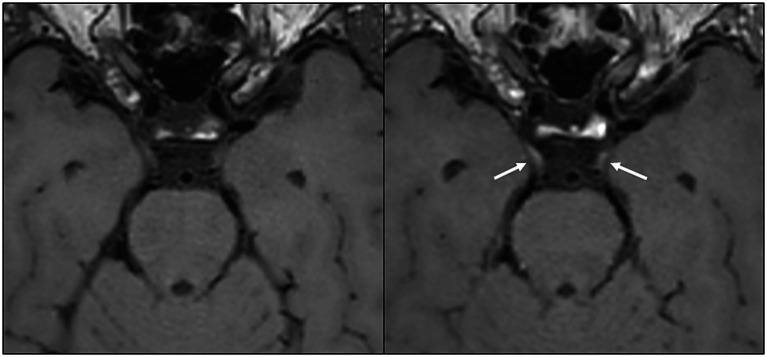
Axial image reconstruction from a black-blood T1 space sequence before (left) and after (right) contrast administration, showing bilateral contrast enhancement in the cisternal portion of the oculomotor nerve (arrows). This MRI finding was present 13.5 months after the onset of COVID-19 in a middle-aged woman who developed a new-onset, persistent post-infectious headache.

Cranial nerve enhancement persisted in all five initial participants at the 12-month follow-up (Figure 2). Additionally, two new participants exhibited enhancement at 12 months, including one who had received contrast at 6 months. Among the 7% (7/94) with persistent CNE, four were hospitalized in the acute phase, including one admitted to the ICU, while the remaining three were outpatients. CSF samples were obtained from four of the participants with CNE, none indicating signs of inflammation (white blood cells <4 × 10^9^/L; mean total protein 0.41 g/L, range 0.36–0.45 g/L).

## 4 Discussion

In this prospective multicenter study involving patients referred for specialist evaluation due to neurological symptoms following COVID-19, WMHs did not differ from healthy controls when evaluated with Fazekas score and lesion count, and the prevalence of CMBs aligned with rates observed in population studies (12). Further, MRI changes occurring between the 6- and 12-month follow-ups were rare. Notably, a subset of patients exhibited signs of inflammation up to 1 year after infection, suggesting prolonged pathophysiological changes.

### 4.1 Vascular changes

WMHs and CMBs are considered indicative of cerebral small vessel disease, and their prevalence increases with age and comorbidities (13). Vascular changes after COVID-19 are extensively studied, with evidence suggesting that persistent inflammation and endothelial dysfunction can increase the risk of vascular complications (14–16). However, the multifactorial nature of potential brain injury in PCC should be acknowledged, including contributions from coagulopathy, microthrombotic events, and demyelination (17, 18). An analogous study observed new punctate hyperintensities on T2 FLAIR sequences conducted 2 months after infection in all participants (19). Our findings do not indicate that these hyperintensities continue to develop beyond 6 months following infection. WMH severity in our cohort aligned with healthy controls and population studies (20, 21), with visual assessments showing no significant change in observed WMHs between 6 and 12 months. This information is relevant for clinical practice. In a scientific context, quantitative measurements could more accurately elucidate disease progression or reversal, recognizing the possible dynamic nature of this process (22), potentially reflecting a broad etiological spectrum.

No new ischemic lesions were detected during follow-up. However, small ischemic changes obscured by existing WMHs cannot be ruled out. Notably, vessel wall enhancement, indicating persistent inflammation in the brain vasculature, was not detected. This absence is important given our use of a sensitive contrast enhanced T1 space black-blood imaging sequence designed specifically to identify such pathological changes at both the 6- and 12-month time points.

CMB prevalence aligned with population norms (12), with few new occurrences during follow-up, remaining within expected ranges (23). The frequency of CMBs is notably higher among ICU patients compared to non-ICU patients in COVID-19 (24, 25), influenced by factors such as critical illness, hypoxia, age, and comorbidities. We observed similar patterns, with hospitalized patients being older and having more comorbidities (Table 1), which correlated with a greater burden of WMH and CMB. The number of CMBs has not been shown to predict cognitive dysfunction in COVID-19 patients (24, 26). Conversely, quantitative MRI assessments reveal grey and white matter alterations potentially linked to long-term cognitive sequelae, with increased cortical thickness and lower fractional anisotropy in specific brain regions correlating negatively with memory performance (27). Such findings suggest the need for monitoring this patient group and further exploration of the subject.

### 4.2 Signs of inflammation

Various brain MRI abnormalities are documented as SARS-CoV-2 complications, including findings consistent with encephalitis and ADEM (28, 29). In our study, findings interpreted as inflammatory or demyelinating lesions surfaced in five patients at 6 months, persisting in most of them at 12 months. Quantitative MRI techniques have been utilized to identify neuroinflammatory and demyelinating cerebral changes over a 10-month period following COVID-19 (30, 31). These studies have detected alterations in grey matter morphometry and white matter microstructure, which show partial recovery and correlate with disease severity and inflammatory markers. The mechanisms and long-term implications of these changes are under investigation and may provide insights into the neurological symptoms seen in PCC. A national register-based study found that hospitalization for COVID-19 was linked to increased risk of developing MS compared to individuals without a COVID-19 diagnosis. Longer follow-up studies are warranted to establish whether a causal association exists (32). Our finding of infrequent longitudinal MRI changes suggests that, in a clinical context, repeated imaging should be reserved for patients who develop new symptoms, whereas clinical follow-up on an individual basis is sufficient for the majority.

Inflammatory signs in relation to COVID-19 detected on MRI include any cranial nerve involvement (33). Contrary to literature on acute/subacute phase of COVID-19, persistent post-infectious cranial nerve enhancement is rarely reported. However, we observed CNE in a small subset of patients using a routine sequence, possibly indicating long-lasting blood-nerve barrier (BNB) integrity alterations. Findings indicating blood–brain barrier (BBB) dysfunction after COVID-19 have been reported. Elevated astrocyte plasma biomarkers peaking 4 months post-hospitalization indicate transiently elevated BBB permeability (34). Dynamic contrast-enhanced MRI findings link ongoing BBB dysfunction to long COVID-associated brain fog (35).

CNE is observed in varied conditions and can persist for extended periods. BNB dysfunction can be observed in the context of neuroinflammation such as in MS, neurosarcoidosis, autoimmune conditions, and following several infections including herpes simplex, varicella zoster, cytomegalovirus, Lyme neuroborreliosis, and tuberculosis (36–38). In six of seven patients in this study, the neuropathy was not clinically apparent, and the CSF showed no signs of inflammation, suggesting that further investigation is needed to comprehend the clinical implications. Subclinical CNE is not uncommon in different diseases (39, 40). This may be attributed to mild inflammation that does not lead to neuronal dysfunction, residual contrast uptake following prior inflammation, or merely increased perineural vascularization. In our cohort, one patient developed CNE more than 6 months after infection, and a similar case has previously been documented (41), in addition to reports of CNE subsequent to COVID-19 vaccination (42). Delayed findings like these should alert us to the possibility of immune-mediated mechanisms.

### 4.3 Strengths and limitations

Our study features a large cohort, scanned at two distinct time points using a comprehensive imaging protocol that includes contrast enhanced sequences. This robust methodology enables a thorough evaluation of cerebral MRI findings and their temporal changes, effectively addressing knowledge gaps in the existing literature. By consistently performing follow-up MRIs at 6-month intervals, we control for time-related variables and increase data comparability. Additionally, unlike many previous studies, we base PCC diagnoses on thorough clinical examinations rather than questionnaires. This combination of high diagnostic accuracy and precise timing of MRI intervals enhances comparability with published results.

The primary limitations of this study stem from its multicenter design. Images were acquired using different MRI scanners with field strengths of 1.5 or 3 T, alongside some variability in MRI protocols, potentially affecting the detection rates of pathologies such as CMBs and contrast enhancement. While larger sample sizes increase statistical power, technical variability may offset this advantage. The majority (80/140) of participants, however, were scanned using the same 3 T MRI scanner at the main recruitment center. Comparing WMHs in the PCC group with a healthy control group was conducted, and similar comparisons for CMBs and contrast enhancement could further strengthen our results.

Our methodology, including lesion count and ordinal or dichotomous scores of WMHs, CMBs, and contrast enhancement, may be insufficient for longitudinal assessments, as they fail to capture subtle changes. More sensitive and quantitative MRI analyses are required to detect fine-scale pathologies. Despite these limitations, our findings provide valuable insights for clinical decision-making regarding neuroimaging indications and methods in the follow-up of PCC patients exhibiting neurological symptoms, enhancing our understanding of how to manage these patients over time.

Clinical research indicates that PCC often follows mild illness (1), and evidence suggests that the severity of the disease during the acute phase may not correlate with microstructural brain abnormalities (27). No clear distinction was observed between hospitalized and non-hospitalized individuals with CNE in our cohort. Comparison with a control group that experienced COVID-19 but did not develop PCC could strengthen the suspected association between imaging findings and PCC. Future research should focus more on the distinct neurological phenotypes of PCC, which may arise from different or interconnected pathophysiological mechanisms, including vascular dysfunction, neuroinflammation, BBB disruption, and autoimmune responses (43).

A clinico-radiological gap exists between prevalent neurological and cognitive complaints and brain findings identified through standard imaging techniques. Advanced, quantitative neuroimaging analyses—not yet established in clinical practice—show potential for detecting subtle neuronal changes, thereby deepening our understanding of underlying pathophysiological mechanisms.

## 5 Conclusion

Pathological brain MRI findings are infrequent in PCC patients with neurological symptoms at 6 months, supporting the notion that MRI primarily serves to exclude differential diagnoses in clinical practice. Contrast-enhanced sequences may help in detecting long-term inflammation. Our findings suggest that repeated imaging should be performed only when new symptoms emerge. These insights will assist in shaping future brain imaging needs and guidelines for diagnosis and follow-up of PCC patients with neurological complaints.

## Data Availability

The raw data supporting the conclusions of this article will be made available by the authors, without undue reservation.
