# Supplementary material for: Brain MRI findings in patients with post COVID-19 condition: frequency and longitudinal changes in a nationwide cohort study
Source: Front Neurol. 2025 Nov 13;16:1662263. doi: 10.3389/fneur.2025.1662263 (PMC12658414; doi:10.3389/fneur.2025.1662263)
Supplement: Supplementary file 1 [file Table_1.docx]

Supplementary Material

# Supplementary Tables

**Supplementary Table 1.** **Magnetic resonance imaging study protocol with intravenous gadolinium contrast agent and 32 channel head coil.**

| **Sequence** | **Acquisition time** | **Voxel size (mm)** | **FOV read (mm)** | **Key parameters** |
| --- | --- | --- | --- | --- |
| T1 MPRAGE | 6:38 | 0.8x0.8x0.8 | 256 | TR/TE/TI (ms) 2400/2,22/1000 |
| T2 SWI tra | 5:28 | 0.3x0.3x1.0 | 230 | TR/TE (ms) 30/20 |
| T1 SPACE sag blood suppressed | 5:04 | 0.4x0.4x0.9 | 217 | TR/TE (ms) 700/12 |
| T2 SPACE FLAIR sag | 6:07 | 0.4x0.4x0.9 | 256 | TR/TE/T1 (ms) 5000/385/1800 |
| TOF 3D 5 slabs | 7:34 | 0.3x0.3x0.5 | 200 | TR/TE (ms) 21/3,43 |
| DWI RESOLVE ax | 2:49 | 1.4x1.4x4.0 | 220 | TR (ms) 5220, b (${s/mm}^{2})$ = 0, 1000 |
| ep2d perf PA | 0:16 | 1.7x1.7x4.0 | 220 | TR/TE (ms) 1360/30 |
| ep2d perf AP | 2:12 | 1.7x1.7x4.0 | 220 | TR/TE (ms) 1360/30 |
| T1 SPACE sag blood suppressed, Gadovist | 5:04 | 0.4x0.4x0.9 | 217 | TR/TE (ms) 700/12 |
| T1 MPRAGE, Gadovist | 6:38 | 0.8x0.8x0.8 | 256 | TR/TE/TI (ms) 2400/2,22/1000 |
| Ax SWI | 5.55 | 0.9x0.9x0.9 | 220 | TR/TE (ms) 27/20 |
| TOF 2D tra^a^ | 0:54 | 0.5x0.5x5.0 | 256 | TR/TE (ms) 20/4,56 |
| ASL strong BS^a^ | 6:06 | 3.4x3.4x3.3 |  | TR/TE (ms) = 4300/36, PLD 2000 ms |
| 3D FLAIR sag  (Skyra/Signa)^b^ |  | Slice thickness (mm) 0.48/1.2 | 245/256 | TR/TE/TI (ms) = 5000/8000 /389/90 / 1800/2071 |

Scan parameters for the imaging sequences. At the main recruitment center, MRI was performed with a Magnetom Prisma 3T MRI system (Siemens Healthineers), Software Version *syngo* MR E11. Contrast agent Gadovist (Bayer Inc.). A similar protocol was made for Philips Healthcare MRI Machines. Certain sequences were available only at the main recruitment center.

^a^The sequence was performed on participants who did not receive an intravenous gadolinium contrast agent.

^b^Controls were scanned on two different 3T MRI scanners, a Skyra system (Siemens Healthineers) with a 64 channel head coil and a Signa system (General Electric Healthcare) with a 32 channel head coil.

**Supplementary Table 2. Interrater reliability of MRI findings.**

| **Variable** | **Coefficient of agreement^a^** | **Raters^b^** |
| --- | --- | --- |
| Fazekas score (0 – 3) (PCC) | 0.93 | Rater LLF vs. Rater OL |
| Fazekas score (0 – 3) (Controls) | 0.81 | Rater LLF vs. Rater ESL |
| CMB (0/1) | 0.77 | Rater LLF vs. Rater OL |
| Contrast enhancement (0/1) | 0.93 | Rater LLF vs. Rater OL |

^a^Interrater reliability was assessed with Cohen’s Weighted Kappa for Fazekas score and Cohen’s Kappa for CMBs and contrast enhancement.

^b^LLF, OL and ESL are abbreviation for specific raters involved in the study.

**Supplementary Table 3. Contingency table of WMH distribution and changes during follow-up.**

|  | **PCC** | **Controls** | ***p*-value^b^** | **Age-adjusted *p*-value^c^** |
| --- | --- | --- | --- | --- |
| **Fazekas score** | n=139 | n=64 | 0.128 | 0.064^d^ |
| 0 | 64 (46.0%) | 21 (32.8%) |  |  |
| 1 | 64 (46.0%) | 38 (59.4%) |  |  |
| 2 | 7 (5.0%) | 5 (7.8%) |  |  |
| 3 | 4 (2.9%) | 0 (0%) |  |  |
| **WMH lesion count, 6 months^a^** | n=120 | n=64 | 0.001 | 0.295 |
| 0 | 56 (46.7%) | 16 (25.0%) |  |  |
| 1-9 | 50 (41.7%) | 31 (48.4%) |  |  |
| 10-20 | 7 (5.8%) | 7 (10.9%) |  |  |
| <20 | 7 (5.8%) | 10 (15.6%) |  |  |
| **WMH lesion count, 12 months^a^** | n=107 | n=62 | 0.002 | 0.256 |
| 0 | 50 (46.7) | 16 (25.8) |  |  |
| 1-9 | 44 (41.1) | 29 (46.8) |  |  |
| 10-20 | 7 (6.5) | 7 (11.3) |  |  |
| >20 | 6 (5.6) | 10 (16.1) |  |  |
| **Change in WMH category^a^** | 2 (2.3%) | 2 (3.2%) | 1.00 |  |
| **Change in WMH count^a^** | 8 (9.1) | 9 (14.5) | 0.310 |  |

^a^MRI at 6 and 12 months after onset of COVID-19 correspond to baseline and 6-month intervals for controls. 88 PCC patients and 62 controls underwent T2 FLAIR imaging at both time points.

^b^p-values were calculated using the Mann–Whitney U test for ordinal variables, and Fisher’s exact test for categorical changes.

^c^Adjusted *p*-value derived from ordinal logistic regression (with logit link) incorporating age as a covariate.

^d^To examine whether the link between group and Fazekas score was due to higher comorbidity among hospitalized patients, a sensitivity analysis excluding these patients was performed using ordinal logistic regression adjusted for age. The unadjusted analysis showed no significant group difference (*p* = 0.128), but post-age adjustment, the *p*-value dropped (*p* = 0.064), indicating a trend toward higher scores in the COVID group than age alone would predict. Excluding hospitalized patients raised the *p*-value to 0.103, suggesting their comorbidities partly explained the primary results.
